# Personal

**Published:** 1907-09

**Authors:** 


### PERSONAL.

Dr. Edward N. Brush, of Baltimore, formerly of Buffalo and editor of this Journal, now superintendent of Sheppard and Enoch Pratt Hospital for the Insane at Towson, a suburb of Baltimore, sailed for Europe, August 17, 1907. Dr. Brush was accompanied by his son Nathaniel, and will attend the International Congress of Psychiatry, Neurology, and Psychology, at Amsterdam, September 2-7, 1907, under a credential of the department of state, as an official representation of the United States. He will return early in October to take up his duties at the hospital, and his son will then resume his work at Johns Hopkins University.

Dr. Walter D. Greene, of Buffalo, formerly health commissioner of this city, has formed a copartnership with Dr. George L. Brown, their offices being established at No. 210 D. S. Morgan Building.

Dr. B. H. Grove, of Buffalo, who went to Europe early in the summer, has been spending some time recently at Vienna. He expects to return and resume his professional work on or about September 1.

Dr. Jane W. Carroll, of Buffalo, was elected vice-president of the medical section of the National Fraternal Congress during its recent session in this city.

Dr. O. Millard, of Flint, Mich., president of the medical section of the National Fraternal Congress, visited Buffalo during the third week in August while the organisation was in session here.

Dr. Magnus A. Tate, of Cincinnati, has been appointed professor of obstetrics at the Miami Medical College in that city.

Dr. W. J. Means, of Columbus, chairman of the judicial council of the Association of American Medical Colleges, spent a few days in Buffalo during August in attendance upon the medical section of the National Fraternal Congress.

---

Dr. A. W. Hengerer, of Buffalo, announces the removal of his office and residence to 301 Genesee street. Hours: 8 to 9, 1 to 2 and 7 p.m. Sundays: 2 to 4 only. Both Telephones.

